# Neuron–Oligodendrocyte Communication in Myelination of Cortical GABAergic Cells

**DOI:** 10.3390/life11030216

**Published:** 2021-03-09

**Authors:** Elisa Mazuir, Desdemona Fricker, Nathalie Sol-Foulon

**Affiliations:** 1 Inserm, CNRS, Paris Brain Institute, ICM, Sorbonne University, Pitié-Salpêtrière Hospital, F-75013 Paris, France; 2 CNRS UMR 8002, Integrative Neuroscience and Cognition Center, Université de Paris, F-75006 Paris, France

**Keywords:** Myelin, oligodendrocytes, oligodendrocyte lineage cells, GABAergic neurons, interneurons, fast-spiking PV^+^ cells

## Abstract

Axonal myelination by oligodendrocytes increases the speed and reliability of action potential propagation, and so plays a pivotal role in cortical information processing. The extent and profile of myelination vary between different cortical layers and groups of neurons. Two subtypes of cortical GABAergic neurons are myelinated: fast-spiking parvalbumin-expressing cells and somatostatin-containing cells. The expression of pre-nodes on the axon of these inhibitory cells before myelination illuminates communication between oligodendrocytes and neurons. We explore the consequences of myelination for action potential propagation, for patterns of neuronal connectivity and for the expression of behavioral plasticity.

## 1. Introduction

Cortical circuit function is shaped by the cellular electrophysiology of different sets of cortical neurons and synaptic communication between them. Most cortical neurons are excitatory, while a minority, about 20%, release the inhibitory neurotransmitter GABA (gamma-aminobutyric acid). Some GABAergic neurons (or interneurons) form synaptic contacts with nearby principal cells, while others also project to more distant target cells. GABAergic signaling generally acts to counter glutamatergic excitation, with specific groups of interneurons fulfilling distinct operations. Interneurons can generate neuronal timing, which enforces temporal precision in excitatory signals. Different GABAergic cells form synapses with distinct regions of the pyramidal cell membrane and so can provide a shunting somatic inhibition, functionally silence dendritic branches or modulate integration by changing the balance of somatic and dendritic excitation.

Glial cells outnumber neurons in the mammalian cortex. They play critical roles in neuronal development and survival as well as in the establishment and regulation of neuronal networks and information processing. They comprise cells of the oligodendroglial lineage as well as astrocytes and microglia. The role of astrocytes and microglial cells in synapse formation and plasticity has been well described in excellent reviews [1,2] and will not be developed here. Oligodendrocytes enhance neuronal function by producing a myelin sheath that surrounds axons. Myelin accelerates action potential conduction and regulates transmission, critical for the coherent arrival of synaptic inputs carried by multiple axons in sensory systems [3,4]. Oligodendrocytes release factors that signal to neurons during myelination and provide metabolic support to axons [5,6,7].

Axons of GABAergic neurons, including local interneurons, as well as pyramidal cells can be myelinated in the cortex and hippocampus of rodents and humans [8,9,10,11]. This review will examine interactions between oligodendrocytes and GABAergic neurons. Nodal proteins cluster on axons of some GABAergic cells before myelination. We will examine signaling between oligodendrocytes and GABAergic neurons as myelination develops and also metabolic signaling between these cell types. The review explores the effects of interneuron myelination on action potential propagation and neuronal connectivity and plasticity.

## 2. GABAergic Neuron Properties

### 2.1. GABAergic Neuron Diversity and Origins

Almost a century ago, Ramon y Cajal described a vast diversity in neuronal morphologies leading him to qualify them as “butterflies of the soul”. He surmised that neuronal heterogeneity was associated with a diversity of function in cortical circuits (Ramon y Cajal, 1923). Our present understanding of the features and functions of cortical neurons, excitatory and inhibitory, derives from techniques including anatomy, electrophysiology and molecular biology (for review see [12,13,14,15,16]. While pyramidal cells are not uniform, GABAergic cells possess a startling diversity. Agreement on how this diversity should be classified remains to be established. In 2008, an exhaustive list of features that differentiate GABAergic neurons was compiled by a group of researchers, the Petilla Interneuron Nomenclature Group [17]. Their nomenclature was based on (i) morphological criteria, including axonal and dendritic form and orientation, (ii) molecular contents, including calcium-binding proteins (calbindin, calretinin, parvalbumin), possible neuropeptide co-transmitters (neuropeptide-Y, vasoactive intestinal peptide, cholecystokinin and somatostatin) and transcription factors and (iii) physiological properties, including firing pattern [17]. A loose classification into three major groups accounts for nearly all cortical GABAergic neurons: parvalbumin-expressing (PV^+^) neurons, somatostatin-expressing (SST^+^) neurons, and ionotropic serotonin receptor-expressing (5HT3aR^+^) neurons [13]. These groups may not be exclusive since some PV^+^ interneurons also express SST, at least transiently [18,19]. The more recent application of single-cell RNA sequencing has provided detailed data on the molecular diversity of GABAergic neurons and how it is correlated with anatomy and physiology [20,21,22,23,24,25,26]. Linking the transcriptional diversity of mature cortical interneurons to the expression of distinct transcription factors led to an estimate of at least 23 GABAergic neuronal types [26]. An alternative classification based exclusively on morphology and physiological properties has identified 68 distinct morpho-electric inhibitory combinations [27]. Dynamic gene regulatory networks including transcription factors determine developmental trajectories and define stable identities for GABAergic cells [16]. Transcriptional profiles of interneurons are suggested to govern synaptic connectivity and the properties of synaptic communication [28].

**Figure 1:**
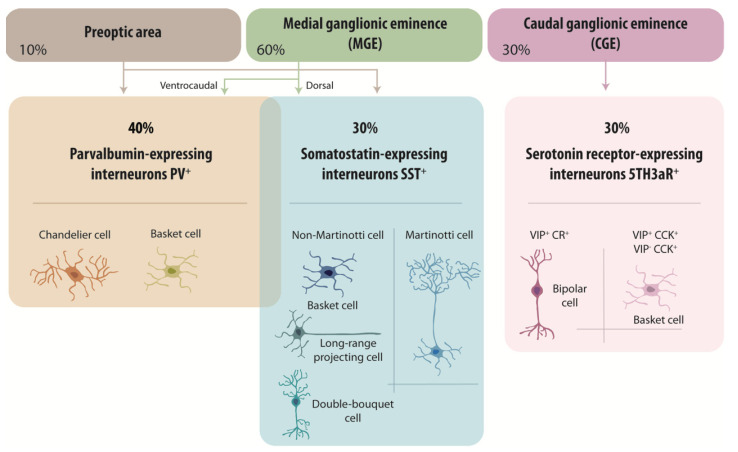
A classification of cortical GABAergic neurons. Three main classes account for almost all cortical GABAergic neurons: parvalbumin-expressing (PV^+^) neurons, somatostatin-expressing (SST^+^) neurons, and ionotropic serotonin receptor-expressing (5HT3aR^+^) neurons. Of the PV^+^ cells, chandelier cells are axo-axonic cells that synapse with the initial segments of pyramidal cell axons. PV^+^ basket cells are the most abundant type of neocortical interneuron. They are fast-spiking cells that innervate the soma and proximal dendrites of pyramidal cells and other interneurons. A small fraction of PV^+^ basket cells also expresses SST. There are two major types of SST^+^ interneuron: Martinotti cells, which innervate pyramidal cell dendrites, and non-Martinotti cells, which include cells that project over long distances. 5HT3aR neurons can be divided into two subgroups based on the expression of the neuropeptide VIP. Different classes of interneurons are generated and specified from spatially distinct progenitor cells in the preoptic area (POA), the medial and caudal ganglionic eminences (medial ganglionic eminences (MGE) and caudal ganglionic eminences (CGE), respectively).

Expression of combinations of spatial and temporal fate determinants during early development govern distinct phenotypes of a remarkable variety of interneurons. Actions of these determinants are initiated in the subpallium, a discrete region of the neural tube in ventral telencephalon, from which cortical GABAergic neurons originate. It develops into lateral, medial and caudal ganglionic eminences (LGE, MGE, CGE), preoptic area (POA) and septum (SE). Progenitors from different domains of the subpallium express different combinations of transcription factors that govern their fate [29]. Cell fate analysis and migration assays indicate that MGE and CGE are major sources of cortical GABAergic neurons [30,31,32], with a lesser contribution from the POA [33,34]. Nearly all PV^+^ and SST^+^ cells migrate from the MGE and POA [33,34,35,36,37], while heterogeneous cell groups, including VIP^+^ and CCK^+^ interneurons, emerge from the CGE [38] (Figure 1).

How does the subpallium generate so many different cell fates? One factor is that progenitors expressing similar transcription factors are spatially restricted to discrete regions of the subpallium. For example, progenitors of the MGE all express the homeodomain transcription factor Nkx2.1 [39,40], but only those located in dorsal MGE express the transcription factor Nkx6.2 while in ventral MGE, Etv1 is expressed [15]. Fate examination indicates that SST^+^ GABAergic neurons tend to migrate from the dorsal MGE while PV^+^ cells originate from more ventrocaudal regions of the MGE [29,41,42,43]. A dorso-ventral sonic hedgehog signaling gradient is suggested to govern this spatial segregation of sites of origin for PV^+^ and SST^+^ cells in the MGE [44]. However, spatial cues may not completely explain fate determination, since clonal studies show individual MGE progenitors can produce both SST^+^ and PV^+^ clones [45,46,47]. Timing seems also to be important: SST^+^ GABAergic neurons are generated during early neurogenesis while PV^+^ cells are generated later [43,48]. This sequence of cell birthdates predicts the “inside out” laminar cortical organization of SST and PV^+^ GABAergic neurons [42,49,50]. Migration along radial glial cells starts when the first cells are generated. Definitive specification, from cues received in migration and at final cortical destination sites, determines local afferent and efferent connectivity, reviewed in [16] (Figure 1).

We note that the same subpallium germinal regions give rise to oligodendrocyte precursor cells (OPCs) as well as about 70% of cortical GABAergic neurons. Similarities in transcriptional architecture associated with this common origin may favor later interactions between neurons and oligodendrocytes [51]. Reports that MGE-derived precursors expressing the oligodendroglial markers nerve/glia antigen 2 (NG2) and Olig2 later differentiate into cortical interneurons adds to the evidence for an overlap [48,52,53]. Furthermore, GABAergic neurons and ventrally-derived OPCs both exhibit high rates of programmed cell death during the first two postnatal weeks in mice [54,55,56]. Recent work has also shown that ventrally-derived interneurons initiate synaptic responses in ontogenetically-related OPCs and that the two cell types form small clusters of cells throughout the mouse somatosensory cortex [57]. These data point to privileged interactions between OPCs and cortical GABAergic neurons.

### 2.2. GABAergic Neuron Functions

GABAergic neurons serve a wide range of cortical functions beyond their classical action to counter neuronal excitation. Notably, inhibitory cells can control the timing of firing in populations of pyramidal cells since local synaptic connectivity is very high (more than 50%) for some GABAergic cell types. Such interactions between inhibitory and excitatory neurons are crucial for the generation of rhythmic activities necessary for cortical information processing. An impaired excitatory/inhibitory balance is linked to neurological disorders, including epilepsy, autism spectrum and schizophrenia [58,59].

PV^+^ interneurons represent about 40% of neocortical inhibitory cells, for review see [14]. They principally consist of fast-spiking basket cells, which synapse with the soma and proximal dendrites of principal cells and other inhibitory cells (Figure 1). PV^+^ cells fire action potentials at high frequencies (>50 Hz at 22 °C and >150 Hz at 34 °C). Chandelier cells are also PV^+^ and these “axo-axonic cells” form synapses selectively with the axon initial segment of pyramidal cells [14,60]. Fast-spiking PV^+^ interneurons possess multiple dendrites of a total length up to 3–9 mm [61,62,63,64]. They receive a high density of inhibitory and excitatory synapses on dendrites and around the soma. For example, CA3 and CA1 hippocampal GABAergic cells are innervated by 16.000–34.000 synaptic terminals, the vast majority of them excitatory [61,62,63,64]. Axon of fast-spiking PV^+^ interneurons arborize very extensively in a local region forming 1000s of distal “en passant” boutons, which contact peri-somatic regions of the pyramidal cell membrane [65]. Action potentials are initiated proximally and propagate with high reliability and higher speeds than in principal cell axons [66,67]. PV^+^ interneuron axons express very high densities of voltage-gated sodium channels, especially Na_v_1.1 and Na_v_1.6 isoforms, which facilitate rapid action potential propagation and repetitive firing at high frequency [68]. Fast, repetitive firing is essential to PV^+^ interneuron functions in cortical and hippocampal circuits. In responses to afferent excitation, interneurons fire before pyramidal cells, as in the feedforward inhibition of the CA1 region when Schaffer collaterals are stimulated [69,70]. Fast-spiking PV^+^ interneurons operate to limit pyramidal cell firing, adjusting their excitability to remain sensitive to weak inputs but not to saturate with stronger stimuli [69,70,71,72,73]. PV^+^ interneurons are also activated by local pyramidal cell activity in feedback circuits, which may underly pattern separation [74,75] and activity sparsification [76].

Nearly 30% of cortical GABAergic neurons express somatostatin (Figure 1) and these SST^+^ interneurons include Martinotti cells and non-Martinotti cells [77]. Martinotti cells are mostly located in layers II/III of the cortex, and project to layer I where they synapse onto distal pyramidal cell dendrites. Equivalent SST^+^ interneurons in the hippocampal also innervate distal pyramidal cell dendrites. These SST^+^ cells represent almost 15% of total GABAergic cortical interneurons [78,79,80,81] and mediate feedback inhibition of pyramidal cells [77,82]. Non-Martinotti SST^+^ cells, which include long-range projection interneurons, double-bouquet cells and some basket cells, form synapses with both pyramidal cells and PV^+^ cells. The axons of long-range SST^+^ interneurons project out of a region of the cortex or hippocampus to innervate functionally distinct brain areas. SST^+^ hippocampal interneurons innervate neurons of the medial septum and entorhinal cortex. These projections are mirrored by a reciprocal back-projection and have been linked to the synchronization of oscillatory activity between distant regions [8,83,84,85]. Both SST^+^ and PV^+^ interneurons participate in the generation of synchronous rhythmic inhibition [86,87,88,89,90] of pyramidal cells at gamma frequencies (30–120 Hz). Gamma oscillations have been linked to cognitive tasks including working memory and attention [91,92].

The remaining 30% of interneurons are immunopositive for 5HT3aR^+^ (Figure 1). These heterogenous interneurons co-express markers including vasoactive intestinal polypeptide (VIP) [13,93], lysosomal marker proteins (LAMP) [27] or synuclein gamma (SNCG) [24]. VIP^+^ interneurons are mainly found in cortical layers II/III and were first thought to innervate only other GABAergic cells (PV^+^ and SST^+^) to mediate disinhibition [94,95]. More recent data shows they also target principal cells [96]. Bipolar VIP^+^ interneurons, co-express the calcium binding-protein calretinin (CR) [97] project an axon into deep cortical layers and fire irregularly. In contrast, multipolar VIP^+^ interneurons are basket cells, co-express the neuropeptide cholecystokinin (CCK) and fire in a regular or bursting pattern. These cells are transcriptionally similar to 5HT3aR^+^ basket cells of layers V and VI, which express CCK but not VIP.

## 3. Oligodendroglial Cells and Their Interactions with Neurons

### 3.1. Oligodendroglial Lineage Cells

Oligodendrocyte precursor cells (OPCs) proliferate and migrate in the central nervous system (CNS) before differentiating into myelin-forming oligodendrocytes [98]. Several intrinsic and extrinsic signals promote the expression of stage-specific markers during maturation, resulting in subgroups of oligodendrocyte lineage cells with distinct abilities to proliferate and migrate, as their morphology changes [99]. This diversity of lineage cells has been explored with single-cell RNA sequencing, anatomy and functional responses to neurons [100] (reviewed by Bostrand and Williams in this issue).

OPCs are small bipolar cells expressing specific markers including the transmembrane proteoglycan NG2, platelet-derived growth factor receptor α (PDGFRα) and the transcription factors Olig1/2 together with the ganglioside A2B5. OPCs have high capacities to proliferate and migrate in early developmental stages [101,102]. During migration, they extend and retract growth-cone-like processes, to sense chemotactic signals such as sonic hedgehog (Shh), bone morphogenic proteins (BMPs) and Wingless-related integration site (Wnt) glycoproteins [99]. OPC processes also survey neighboring cells by succinct contacts invariably followed by a retraction. This self-avoidance mechanism underlies the maintenance of a rather uniform spacing between OPCs in the brain and spinal cord [103]. Precursor cells remain abundant in the adult, representing 5–10% of cells, and maintain the potential to generate new oligodendrocytes in response to environmental cues [104].

During early postnatal life, some OPCs exit the cell cycle and differentiate into immature, pre-myelinating, oligodendrocytes. NG2 and PDGFRα expressions decrease [105] while sulfatide (O4) and glycolipid galactocerebroside (GalC) expression begin [106]. Morphological changes are initiated, as cells arborize extensively with processes that “look for” axons to myelinate [107]. Pre-myelinating oligodendrocytes mature over several days, expressing myelinating molecules including myelin basic protein (MBP), proteolipid protein (PLP) and myelin-associated glycoprotein (MAG) [108]. As they wrap around axons, cells arrive at the end-point of the lineage: myelinating oligodendrocytes expressing the myelin/oligodendrocyte glycoprotein (MOG) [109].

Oligodendrocyte lineage cells express chondroitin sulfate proteoglycans (CSPGs), including Brevican, Versican isoform V2, Phosphacan and NG2, as well as the glycoproteins Tenascin-R [110,111,112,113,114,115], and Bral1 [116]. These molecules are integrated in a complex with hyaluronic acid, a key component of the brain extracellular matrix (ECM) [117,118]. The ECM forms a dynamic perisynaptic and axonal matrix, which surrounds neurons and glial cells and may participate in plastic, adaptive CNS processes [119,120]. The ECM is modified by matrix metalloproteinases in an activity-dependent manner under the actions of neurons and glial cells [121,122]. We note that astrocytes and neurons also produce ECM proteins with distinct splice variants and glycosylation profiles.

### 3.2. Oligodendroglial Cell Interactions with Neurons

**Figure 2:**
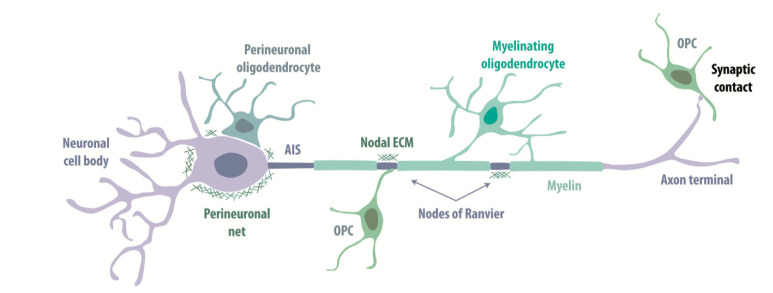
Oligodendroglial cells interact with different neuronal compartments. Schematic representation of interactions between oligodendrocyte lineage cells and CNS neurons. Neuronal cell bodies are illustrated as surrounded by perineuronal oligodendrocytes and extracellular matrix (ECM)-forming perineuronal nets (which are specific for PV^+^ cells). Myelinating oligodendrocytes wrap axons with myelin, leaving small unmyelinated nodes of Ranvier. Nodes are enriched in Na^+^ channels, oligodendroglial-derived ECM and contacted by oligodendrocyte precursor cells (OPCs). Both excitatory and inhibitory neurons make synaptic contacts with OPCs.

Bidirectional interactions between neurons and oligodendroglia are crucial for cortical circuit function. OPCs sense excitatory or inhibitory cell firing by distinct but incompletely understood mechanisms, as described in the review of Habermacher [123]. Oligodendrocytes myelinate axons of both glutamatergic and GABAergic neurons, and fulfill distinct functions in interactions with other neuronal compartments (Figure 2).

#### 3.2.1. Axon Myelination

Myelin corresponds to compacted layers of plasma membrane extensions that wrap spirally around axons. Myelinating elements in the peripheral nervous system are Schwann cells, which form a single myelin sheath around each axon. Oligodendrocytes in the CNS form up to 50 sheaths around multiple axons [124]. The insulating properties of myelin enable rapid, precise action potential propagation over long distances [4,125]. Myelin sheath around an axon is periodically interrupted by nodes of Ranvier, small domains highly enriched in voltage-gated Na_v_ channels, which boost action potentials. Different aspects of myelination for excitatory and inhibitory cortical cells are described in Section 4. Myelinating oligodendrocytes also provide metabolic support including the export of lactate to neuronal axons [5,6,7], see also the review of Tepavcevic on oligodendroglial energy metabolism and (re)myelination in this issue.

#### 3.2.2. Perineuronal Interactions

Perineuronal oligodendrocytes, or satellite oligodendrocytes, in deep cortical layers preferentially surround the soma and basal dendrites of glutamatergic neurons [126,127]. They are less frequently associated with GABAergic neurons [126]. Satellite oligodendrocytes form compact myelin and act to limit the excitability of their host neurons by rapidly buffering K^+^ after firing [127].

#### 3.2.3. Nodal Interactions

Clustering of nodal proteins during myelination depends on interactions with oligodendrocytes (see Section 4.3). At nodes of Ranvier, chondroitin sulfate proteoglycans (CSPGs; including Brevican, Phosphacan and Versican V2, associated with Tenascin-R and Bral-1) form polyanionic molecular complexes that help stabilize nodal structures [113,128,129,130]. These complexes have a high affinity for cations and may prevent Na^+^ diffusion at nodes and so accelerate conduction. ECM interactions with cell adhesion molecules are suggested to localize nodal clusters during initial assembly (see Section 4.3) and also contribute to stabilizing CNS nodes [129,131,132].

Ultrastructural analyses provided the first evidence for interactions of other types of the glial cell at nodes of Ranvier [133,134,135]. Astrocyte processes may participate in potassium buffering at the nodal gap [136,137]. Recent work shows microglial cells preferentially contact axons at nodes of Ranvier, and contact probability is enhanced by K^+^ released at the nodes by neuronal activity [138]. Oligodendrocyte precursor cells also contact nodes of Ranvier, but their role remains elusive [137]. The presence of distinct cell types indicates that nodes of Ranvier constitute a critical site for interactions between glia and neurons.

#### 3.2.4. Perineuronal Nets

The structure and composition of ECM at CNS nodal sites are similar to that of perineuronal nets (PNNs) which ensheath the soma and proximal dendrites of PV^+^ inhibitory neurons [120]. PNNs are suggested to stabilize synaptic connections and so control long-term plasticity. It is notable that PNNs with PV^+^ basket cells are formed during post-natal development as the critical period ends. At this point, sensory experiences initiate plasticity in neuronal circuits less effectively. Critical period plasticity returns when ECM is removed enzymatically by Chondroitinase ABC, suggesting that PNNs act as a brake on experience-dependent plasticity [139,140,141]. PNNs may then protect interneurons from sensory over-activation and stabilize cortical networks [142,143], even at the cost of reduced cortical plasticity and deficits in adult skill acquisition [142].

### 3.3. Effects of Oligodendrocyte Lineage Cells on Synapses

Work on how glial cells affect neural circuit development has been greatly facilitated by the ability to purify and culture neurons in isolation. Twenty years ago, the laboratory of Ben Barres developed glia-free retinal ganglion cell (RGC) cultures [144] and showed the formation of functional excitatory synapses was enhanced when astrocytes were present in co-cultures [145,146,147]. Subsequent work showed that the astrocyte conditioned medium enhances excitatory synaptogenesis [1]. OPCs or oligodendrocytes have been shown to regulate neuronal physiology using similar approaches. Signaling is independent of myelin and communication is bi-directional. Furthermore, OPCs make functional synapses with both excitatory and inhibitory neurons [123,148,149]. OPC secretion of micro-vesicles containing proteins with trophic, modulatory and neuroprotective actions contributes to the homeostasis of neurotransmission [150,151,152,153,154,155].

Oligodendrocytes and OPCs participate in a bi-directional regulation of neurotransmission. Neuronal activity cleaves the NG2 ectodomain on the OPC membrane to release an extracellular domain, which in turn modulates NMDAR-dependent long-term potentiation in pyramidal cells [150]. OPC ablation induces a deficit in glutamatergic signaling by cortical pyramidal cells, which seems to be mediated via reduced secretion of the fibroblast growth factor 2 (FGF2) by NG2 cells [156]. Mature oligodendrocytes secrete the brain-derived neurotrophic factor (BDNF) which modulates glutamate release from excitatory synapses [152]. Mature cells also affect glutamate metabolism via the enzyme glutamine synthetase [157]. The effects of oligodendrocyte expression of glutamine synthetase varies between brain sites possibly due to regional specialization. While the role of astrocytes in glutamate uptake is well established, further work is needed to define whether glutamate and glutamine are exchanged directly via oligodendrocyte transporters or indirectly by astrocyte intermediaries.

The recent production of pure cultures of GABAergic neurons will advance understanding of how these cells are affected by glia secreted factors [158]. Pure cultures are based on cell sorting of fluorescent GABAergic neurons [159] from VGAT-Venus- Wistar rats [160]. In this way, Turko et al. (2019) showed glial-secreted factors influence the growth and survival of both inhibitory and pyramidal cells and that glial factors are needed for the formation of excitatory but not inhibitory synapses [161]. However, the identity of the glial cells was not clearly defined. Our group recently attempted to correct this deficit by work on interactions between GABAergic neurons and factors secreted selectively by oligodendroglia [162]. Electrophysiological and transcriptomic analysis of single GABAergic neurons, revealed that glial cell presence enhances action potential discharge and excitatory post-synaptic potentials (EPSPs) received by GABAergic neurons [162]. Specific changes in transcripts for ion channels, transporters and synaptic markers were induced in glial cell co-cultures and adding oligodendrocyte conditioned medium [163] to purified GABAergic cell cultures partly recapitulated these changes. Conditioned medium also increased axonal length and dendritic arborizations [112,162]. BDNF, a key regulator of interneuron development [164], is a possible candidate as one of the oligodendrocyte secreted factors.

## 4. Myelination of GABAergic Neurons

### 4.1. Identification and Localization of Myelinated Axons

The organization of myelinated axons and nodes of Ranvier in vertebrate CNS underlies rapid, precise conduction of action potentials [4,125]. Myelinated fibers are not homogeneously distributed—some regions contain more myelin than others. Heavily myelinated regions were originally termed white matter, as opposed to grey matter, since lipid-rich myelinated axons appeared white to the naked eye. Axons in neocortical white matter have traditionally been associated with pyramidal cell axons projecting over long distances to form synapses with neurons in distant cortical areas or subcortical regions. The axonal myelination of pyramidal cells with somata in superficial cortical layers is often discontinuous with long unmyelinated segments. In contrast, axons from pyramidal cells of deep layers are typically densely myelinated throughout their trajectory [165]. This organization is correlated with an increased density of mature oligodendrocytes in deeper cortical layers [165].

Axons of GABAergic cortical neurons were first thought to be unmyelinated, possibly since they typically project for only short distances to make local connections. Myelin was first shown to be associated with GABAergic cell axons in electron microscopy studies of cat visual cortex in the 1980s [166,167]. Subsequent work on the myelination of rodent and primate inhibitory cells highlights strong myelination of GABAergic axons in mouse cortex [9,11,168,169,170,171,172], and hippocampus [8,11], in the rat medial septum [83] and entorhinal cortex [173] as well as in human cortex [10,11,169]. One group of inhibitory cells with myelinated axons are the hippocampal SST^+^ long-range projection inhibitory cells that innervate the septum or entorhinal cortex [8]. However, the vast majority of myelinated GABAergic axons are made by fast-spiking PV^+^ interneurons. VIP^+^ and locally-projecting SST^+^ interneurons are rarely and sparsely myelinated [9,11,170]. Myelination of PV^+^ cell axons varies significantly between cortical regions. Array tomography and electron microscopy analysis indicate that myelinated PV^+^ cell axons represent almost 50% of the myelin content in layers II/III of the mouse somatosensory cortex [9]. The fraction can reach 80% in the CA1 region of the mouse hippocampus [11]. In the human cortex, however, with a lower density of synaptic profiles than in the mouse cortex [174] the density of myelinated GABAergic axons is mostly lower than in the mouse cortex, except in the superficial layer I [10].

### 4.2. Characteristics of Myelinated GABAergic Axons

How does this subtype-specific myelination of fast-spiking PV^+^ GABAergic neurons arise? Axonal diameter is known to be a major factor regulating myelination [175,176,177,178]. Reports suggest the diameter of myelinated inhibitory axons is larger than that of pyramidal cell axons [8,9,83,176]. With a similar myelin thickness, this implies a higher ratio between the inner and the outer diameter of the myelin sheath (g-ratio) [9]. Genetic manipulations to increase the size of somata and axons, increased myelin deposition on axons of PV^+^ interneurons from mouse prefrontal cortex [169]. Myelination was also increased in similar experiments on SST^+^ interneurons which originally had thinner and largely non-myelinated axons [169]. These data emphasize that axonal morphology shapes myelination.

**Figure 3:**
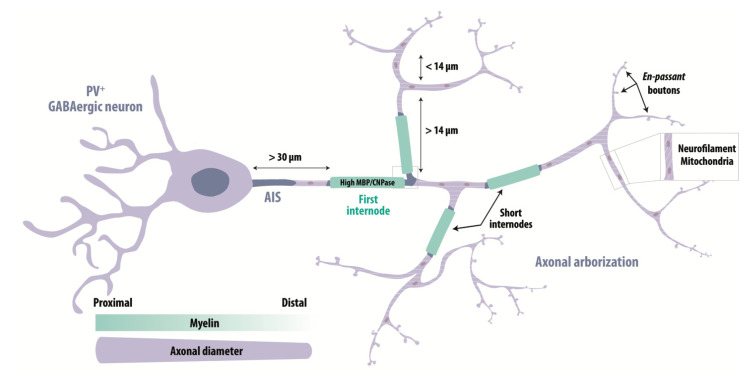
Characteristics of myelinated PV^+^ interneurons. The vast majority of myelinated GABAergic neurons are fast-spiking PV^+^ interneurons. Myelination is most strong along proximal axonal segments, with larger axonal diameters, and diminishes gradually in more distal and thinner axons, which are enriched in “en passant” boutons. The first internode is located at ~30 μm from the site where the axon emerges from the soma. Internodes are segmented by axonal branch points at distances of at least 14 μm. Compared to excitatory neurons, the myelin of GABAergic cells is enriched in CNPase and MBP and axons contain many mitochondria and neurofilaments. GABAergic internodes and nodes are shorter than those of excitatory neurons.

The myelination of PV^+^ GABAergic neurons is most strong for proximal axonal segments, where the axonal diameter is large, and decreases for thinner distal axons [11,169,171] (Figure 3). The first internode is stereotypically located at ~30 μm from the origin of the axon and more distal internodes are segmented by branch points at a minimal separation of ~14 μm [11,169]. Nodes of Ranvier and internode distances are shorter for GABAergic than for pyramidal cells [9]. Myelin composition also differs between excitatory and inhibitory axons: levels of myelin proteolipid protein (PLP) are similar, but myelin basic protein (MBP) is 20% higher in GABAergic axons [9]. Cytoskeletal analysis shows myelinated GABAergic axons are enriched in neurofilaments while excitatory axons contain more microtubules [9]. These results have been confirmed for the human neocortex, where GABAergic axons are enriched in mitochondria as needed to sustain high energy demands of PV^+^ cells [10]. Moreover, myelin is enriched in 2′,3′-cyclic nucleotide 3′-phosphodiesterase (CNPase), a major component of cytoplasmic channels ensuring the connection of oligodendroglial cell body with the myelin sheath and the axonal compartment [10,125] (Figure 3). Differences between myelin of GABAergic and pyramidal cells also extend to remodeling during adaptive responses. Myelinated axons of callosal excitatory projection neurons and PV^+^ interneurons from cortical layer II/III were compared in an in vivo two-photon imaging study of PLP-eGFP mice [179]. During adaptive changes induced by monocular deprivation, myelin of PV^+^ interneurons showed balanced elongations and contractions while myelin of excitatory neurons tended to display elongations alone [179].

Lastly, different structural and molecular properties of distinct subsets of GABAergic cells may influence myelination. Patterns of myelin deposition along inhibitory cell axons during development differ for SST^+^ and PV^+^ interneurons of the mouse visual cortex [170]. These heterogeneities may reflect distinct codes for communication between neurons and oligodendrocytes. Zonouzi and colleagues recently showed that single oligodendrocytes exhibit different patterns of axonal targets [170]. Some oligodendrocytes myelinated only inhibitory cells, some myelinated excitatory neurons and others displayed no bias. While molecular substrates are unclear, mature oligodendrocytes are highly heterogenous [100] and distinct neuronal cues may govern their choice of target cells.

### 4.3. Prenodes Are Formed before Myelination of Hippocampal GABAergic Neurons

The assembly of nodes of Ranvier depends on interactions between oligodendrocytes and neurons [132,180,181]. Clustering of nodal proteins during myelination has been attributed to three, possibly complementary, mechanisms [129]: (i) via formation of paranodes, critical regions where axons interact with myelin and act as a barrier to membrane movements of nodal proteins, (ii) through interaction with extracellular matrix proteins expressed by oligodendroglial lineage cells and nodal Nfasc186 and (iii) by interactions with axonal cytoskeletal scaffolds. Some evidence suggests the mechanisms vary for different types of neuron. Factors secreted by oligodendrocytes can cluster Na_v_ channels on retinal ganglion cells without direct contact with an axon [182,183]. A role for secreted oligodendrocyte cues has been confirmed for the formation of clusters, including Na_v_ channels, Nfasc186, NrCAM and Ankyrin G, at prenodal structures before SST^+^ and PV^+^ GABAergic cell axons are myelinated [112,184]. Time-lapse live imaging of fluorescently tagged markers suggests nodal proteins preassemble before targeting GABAergic cell axons in hippocampal cultures [185]. Mass spectrometry analysis of oligodendrocyte conditioned medium showed the clustering cues consist of Contactin-1 associated with the extracellular matrix proteins Tenascin-R and Phosphacan [112]. Clusters persist and so may participate in node formation by acting as localization signals to guide myelin deposition [185]. We note that hippocampal pyramidal neurons do not form prenodes suggesting that different mechanisms operate during myelination of hippocampal pyramidal cell axons [132,184].

## 5. Myelin, Axonal Conduction and Neural Circuit Function

### 5.1. Determinants of Action Potential Propagation along Myelinated Fibers

Axons convert synaptic inputs into outputs as action potentials are initiated and propagate to synapses where they trigger transmitter release [186]. Conduction velocities depend on temperature, axonal diameter and Na_v_ channel density. The insulating properties of myelin accelerate propagation and clusters of voltage-gated Na_v_ channels at nodes of Ranvier boost velocity, underlying a saltatory form of conduction [187,188,189,190]. Recent work suggests periaxonal and paranodal submyelin spaces may form a second conducting pathway. Cohen et al. [190] used electron microscopy, fast voltage-calibrated optical records from nodal and internodal sites and computational modeling to propose a double cable model for conduction by myelinated neocortical pyramidal axons.

Theoretical studies show that conduction velocity in myelinated axons is linearly proportional to axonal diameter [191]. Conduction also depends on myelin sheath thickness and internodal length [192,193] both of which are linearly related to axon diameter. The size and structure of nodes of Ranvier, as well as Na_v_ channel density, also influence conduction speed [132,194,195,196,197]. Pathological conditions, alter these parameters and so degrade axonal conduction [180]. Our work suggests that clustering of Na_v_ channels at prenodes accelerates conduction, representing another action of oligodendrocytes to speed propagation before myelin is deposited [184]. In addition, hippocampal inhibitory axons express distinct K_v_ channel subunits with different axonal distributions than those expressed by pyramidal cells. K_v_1.2 is selectively enriched all along the axons of hippocampal SST^+^ and PV^+^ cells with prenodes before myelination proceeds [198] and may contribute to regulating firing during development. K_v_1 channels are then progressively enriched at the juxtaparanodes of myelinated axons (see the review of Pinatel and Faivre Sarrailh on assembly and function of the juxtaparanodal K_v_1 complex in this issue), where they contribute to internodal resting potential and act to prevent repetitive firing [199,200]. Specific expression of slowly activating K_v_3.1 and K_v_3.2 channels by PV^+^ axons, combined with fast-inactivating Na^+^ channels, assures high-frequency axonal firing at a low energetic cost [201,202]. K^+^ conductances at nodes of Ranvier are mediated by leak-type channels, identified as TRAAK and/or TREK1 [203,204], and by slowly opening K_v_7.2/K_v_7.3 channels [205].

### 5.2. Effects of Myelination on GABAergic Neurons

Fast-spiking PV^+^ GABAergic interneurons have a key role in local cortical circuits and the speed and reliability of action potential conduction are critical to their functions. The fast-firing, fast signaling phenotype of PV^+^ cells depends on high axonal Na^+^ channel densities [68]. Myelin may provide metabolic support for the high energy needs of PV^+^ cells during sustained high-frequency activities. Two recent studies have asked how myelination affects axonal conduction and the reliability of neurotransmission by GABAergic neurons [171,172]. Micheva and colleagues showed myelination increases conduction velocity in axons of mouse cortical PV^+^ cells, by comparing latencies between interneuron firing and inhibitory post-synaptic currents (IPSCs) and using array tomography images to trace the length and myelination profile of individual axons [172]. The data suggest that increasing myelination and larger axonal diameters accelerate conduction and support temporally precise synaptic interactions. Benamer and colleagues used transgenic mice where myelination defects were induced in PV^+^ cells by inactivating the γ2 subunit of GABA_A_ receptors in OPCs to disrupt PV^+^ cell communication with OPCs [171]. Myelination was severely perturbed in these mutants and was associated with a reduced PV^+^ cell firing, suggesting inhibitory cell maturation was compromised [206]. The strength of feedforward cortical IPSCs was reduced and latencies were increased, consistent with simulations based on slower conduction for dysmyelinated axons [171,196]. These myelination defects for PV^+^ cells of barrel cortex were associated with degraded texture discrimination [171] showing behavioral consequences of dysfunction in cortical inhibitory circuits due to the loss of myelin.

Basket cell axons are characterized by extensive branching with numerous *en-passant* boutons [14]. In mutant *Shiverer* mice, which are deficient for MBP and lack compact myelin [207], basket cell bouton density increases and synapses are located more proximally [11], suggesting myelination influences synapse formation. Cortical feedforward inhibitory circuits have been shown, by 3D reconstructions of multiple electron microscopy sections, to involve thick and highly myelinated interneuron axons, which mediate a precisely timed inhibitory control of pyramidal cell firing [172,173].

### 5.3. Contributions of Myelination to Sensory Processing and Learning

Myelination tunes axonal conduction for precise spike-timing, and so can optimize the synchronous arrival of afferent activity at synaptic relays [3,4,208]. This effect is crucial in the auditory system, where sound localization is computed from time differences, in the sub-millisecond range, between signals from two ears [209]. This sound localization circuit involves nucleus magnocellularis (NM) neurons in birds or neurons of the cochlear nucleus in the mammalian brainstem, which signal bilaterally to the ipsi- and contralateral nucleus laminaris in birds or the medial superior olivary nucleus (MSO) in mammals. Seidl et al. (2010) have shown that axon diameter and internode length vary significantly greatly between ipsi- and contra-lateral branches of NM axons [210]. Modeling suggests these differences operate to adjust conduction speeds to compensate for different axonal lengths [210]. Data on conduction velocities confirms that they are adjusted in the two collaterals to optimize discrimination of differences in timing and sound localization [211].

Neurons in the medial nucleus of the trapezoid body (MNTB), form a distinct part of the sound localization circuit, receiving excitatory inputs from globular bushy cells (GBCs) of the contralateral cochlear nucleus via giant calyx of Held synapses. They project inhibitory signals to binaural comparator neurons in the medial and lateral superior olive (MSO and LSO respectively). Internodal length, internodal axon diameter and node diameter for each GBC axon, all change systematically with distance from the calyx of Held [195]. Computer simulations suggest these graduated changes are essential to minimize conduction delay [195]. These pathway specializations are detected in auditory circuits of the gerbil, which does compute inter-aural time differences, but absent in those of mice, which does not compute such differences. These data suggest axonal myelination is optimized to specific temporal processing requirements of different species [212].

The influence of myelin on the reliability and timing of firing at the calyx of Held synapse [213], has been examined by Kim and colleagues in *shaker* mutant rats, which lack compact myelin, due to a spontaneous genetic deletion of the myelin basic protein (MBP) [214]. Comparison with wild-type animals indicates myelination is crucial for precise presynaptic action potential firing during high-frequency stimulation [214,215]. It enhances the reliability of post-synaptic firing and promotes the precise timing of sound signals in the ascending auditory system [214]. Oligodendrocytes also influence transmitter release at the calyx of Held. Ca^2+^ transients in oligodendrocytes release BDNF, which enhances glutamate release [152]. Moore and colleagues showed impaired metabolic support from myelinating oligodendrocytes also affect auditory processing [216]. They compared auditory brainstem potentials and multiunit activity in the auditory cortex in dysmyelinated mice and in animals with a normal myelin profile but with a deleted monocarboxylate transporter 1 (MCT1 or *SLC16A1*). This transporter mediates metabolic support from oligodendroglia [6]. When neurons fired repetitively, either the reduced metabolic support or the absence of myelin induced conduction failure and affected temporal processing [216]. These data suggest that export of lactate from oligodendrocytes to axons by MCT1 may be critical to maintain repetitive firing.

Can the structure of axonal myelin be adjusted to optimize conduction velocity and synaptic transmission? Communication between excitatory neurons and oligodendrocytes is now known to shape myelination and circuit maturation during experience- or learning-induced tasks in adults [4,123,217,218,219]. New myelin is formed and existing internodes are also remodeled, as well as the width of periaxonal space and the length of node of Ranvier [197,220,221]. These parameters adjustment alters action potential propagation and contributes to promote coincident arrival of synaptic inputs from multiple axons in target regions and improve the fidelity of signal transmission. Neuronal activity also regulates PV^+^ cell myelination. Selective stimulation of cortical PV^+^ cells using the DREADD technique enhances axonal branching and increases myelination [168]. GABAergic cell morphology is important since it determines where myelin is located, or added during de novo myelination of poorly myelinated cells [169]. Adaptive myelination of PV^+^ cells, which innervate large numbers of pyramidal cells, may enhance rhythmic population activities. During the adaptive remodeling of PV^+^ cells, myelination profiles are specific to each cell [179].

## 6. Conclusions

A better comprehension of signaling between oligodendrocyte lineage cells and neurons is central to improve our knowledge of how oligodendrocytes and myelination shape brain circuit maturation. Here we have reviewed their interactions with GABAergic neurons and the functional consequences for inhibitory cell activity, synaptic inhibition, connectivity and optimization of inhibitory circuits. Pathological changes in this dynamic dialog between GABAergic neurons and oligodendrocyte lineage cells may contribute to some CNS psychiatric disorders [222,223]. Recent work on post-mortem tissue also suggests inhibitory cells of the motor cortex may be selectively vulnerable to secondary, progressive demyelinating diseases such as multiple sclerosis [224]. Dissecting mechanisms of bi-directional communication between oligodendroglia and their precursors and GABAergic cells will improve understanding of such vulnerabilities and help develop better therapies for neurological disorders.

## Data Availability

Not applicable.
